# The T-DBSCAN Algorithm for Stopover Site Identification of Migration Birds Based on Satellite Positioning Data

**DOI:** 10.3390/biology14030277

**Published:** 2025-03-07

**Authors:** Xinwu He, Xiqun Liu, Jiajia Liu, Youwen Li, Zhenggang Xu, Ping Mo, Tian Huang

**Affiliations:** 1 Hunan Engineering Research Center of Ecological Environment Intelligent Monitoring and Disaster Prevention and Mitigation Technology in Dongting Lake Region, College of Information and Electronic Engineering, Hunan City University, Yiyang 413000, China; he_xinwu@163.com (X.H.); qwqq5054@gmail.com (X.L.); uy35342@163.com (J.L.); liyouwen358@gmail.com (Y.L.); xuzhenggang@nwafu.edu.cn (Z.X.); moping2021@huas.edu.cn (P.M.); 2 College of Forestry, Northwest A & F University, Yangling 712100, China; 3 College of Life and Environmental Sciences, Hunan University of Arts and Science, Changde 415000, China

**Keywords:** habitat identification, clustering algorithms, time complexity, spatial-temporal random partitioning, bean goose

## Abstract

With the acceleration of social development and urbanization, birds’ natural habitats have been greatly disturbed and threatened. Satellite tracking technology can collect much bird activity data, providing important data support for habitat protection research. However, satellite data are usually characterized by discontinuity, extensive periods, and inconsistent frequency, which challenges cluster analysis. Habitat research frequently employs clustering techniques, but conventional clustering algorithms struggle to adjust to these data features, particularly when it comes to time dimension changes and irregular data sampling. T-DBSCAN, an enhanced clustering algorithm, is suggested to accommodate this intricate data need. T-DBSCAN is improved based on the traditional DBSCAN algorithm, which combines a quadtree structure to optimize the efficiency of spatial partitioning and introduces a convex hull algorithmic strategy to perform the boundary identification and clustering processing, thus improving the efficiency and accuracy of the algorithm. T-DBSCAN is made to account efficiently for the uniformity of data sampling and changes in the time dimension. Tests demonstrate that the algorithm outperforms conventional habitat identification accuracy and processing efficiency techniques. It can also manage large amounts of discontinuous satellite tracking data, making it a dependable tool for studying bird habitats.

## Simple Summary

In this study, the challenge of studying bird habitats using satellite tracking technology, which collects valuable data on bird movements but is often irregular and incomplete, was addressed. This data type can make it difficult to analyze and identify important bird habitats accurately. To solve this, the study proposes an enhanced clustering algorithm called T-DBSCAN, which builds on traditional methods by using a quadtree structure to organize spatial data, a technique for identifying boundaries more accurately. T-DBSCAN also considers both the uneven timing of data collection and the changing behavior of birds over time. This study validates the effectiveness of the T-DBSCAN algorithm by analyzing the multi-year migration trajectories of a single bean goose (*Anser fabalis*) as a case example. The study shows that T-DBSCAN is more efficient and accurate than conventional methods, allowing for better identification of bird habitats. This improvement will be valuable for conservation efforts as it can handle large and irregular satellite tracking data, helping researchers better understand and protect the places where birds live. This tool can ultimately support efforts to protect wildlife and ensure the health of ecosystems.

## 1. Introduction

About 40% of the world’s total bird species perform migration, so bird migration is a relatively common phenomenon [1]. As science and technology have advanced in recent years, the study of bird migration has made significant strides in tracking devices and data analysis [2], particularly satellite tracking technology, which is a crucial tool for studying bird migration because it provides unprecedentedly accurate data for obtaining information on migration paths and stopover habitats [3]. For example, Gschweng et al. used satellite telemetry to study the migration routes of Eleonora’s falcons and found that they have highly individualized path selection [4]. Weimerskirch et al. analyzed the impacts of global climate change on the migration paths, foraging behaviors, and breeding success of albatrosses based on satellite tracking technology [5]. Both et al. investigated the relationship between avian migration and the climate cycle through satellite tracking and revealed the relationship between bird migration and the climate cycle [6]. Satellite tracking can obtain a large amount of data on bird migration paths and habitats, which offers rich resources for examining the selection and evolution of bird habitats.

Bird migration frequently entails a stopover on the way rather than a continuous flight, and the migration always depends on the stopover habitat along the route. Fusani et al., for instance, examined the migration of three species of European finches and found that the birds would pause their journey depending on their physical state [7]. Mikula et al.’s study on the migratory strategies of plovers and sandpipers showed that small-sized species needed more stopovers along the migration route from East Asia to Australasia, which reflected the necessity of multiple stops during long-distance migrations [8]. When discussing the ecological and evolutionary functions of stopover in migratory birds, Schmaljohann et al. pointed out that stopover has the significance of replenishing energy, recovering physiological functions, and escaping from unfavorable environments, highlighting the importance of stopover sites [9]. The habitats at breeding or wintering sites are not the same as the stopover sites during migration, and the habitats during migration are more vulnerable to environmental changes. For example, Li et al. studied the dynamic response to environmental changes during the migration of East Asian white-fronted geese. They pointed out that environmental changes during migration would lead to changes in their stopover sites [10]. Xu et al. studied the migratory phenology of East Asian migratory birds wintering at Poyang Lake. They found that climatic factors, such as local temperatures and the El Niño Southern Oscillation (ENSO), would affect migratory birds’ migration time and stopover sites [11]. Horton et al. found that nighttime artificial light was the primary predictor of birds’ migratory stopover density. This suggests that anthropogenic environmental factors such as light pollution can also shift birds’ stopover sites on their migratory journeys [12]. At the level of data analysis, cluster analysis techniques are widely used to study bird migration paths and stopover habitats. The categorization of satellite data by standard clustering methods such as K-means and Clarans clustering algorithms [13] can help to identify the locations of bird stopover habitats and their distribution patterns. For example, Li Huixia et al. studied the distribution pattern of egrets in Zhejiang Province through the K-means clustering method and drew their migration roadmap [14].

The DBSCAN (Density-Based Spatial Clustering of Applications with Noise) hierarchical clustering algorithm is an important method for exploring the spatial distribution characteristics of bird habitats [15]. However, satellite tracking is often affected by the environment, weather, and the adjustment of the research purpose, resulting in data with temporal discontinuity, uneven quantity, and periodic spatial repetition, and there are certain limitations in habitat identification analysis using conventional clustering algorithms, which are prone to identification errors and omissions. To address this issue, this study suggests a clustering algorithm that integrates temporal factors—Time-DBSCAN—based on the traditional DBSCAN algorithm. By adding the weight parameter of the temporal dimension, the algorithm dynamically modifies the similarity determination criterion in the clustering process, effectively overcoming the difficulties caused by discontinuous data and inconsistent frequency and increasing the accuracy of the analyzing bird migration routes and habitats. This study investigates interannual habitat utilization patterns through long-term tracking of a bean goose (*Anser fabalis*) individual’s migratory behavior, proposing a novel methodological framework for animal migration ecology research. The proposed approach thereby enhances the protection of the natural environment and bird survival by refining habitat analysis precision.

## 2. Materials and Methods

### 2.1. T-DBSCAN Algorithm

**Figure 1:**
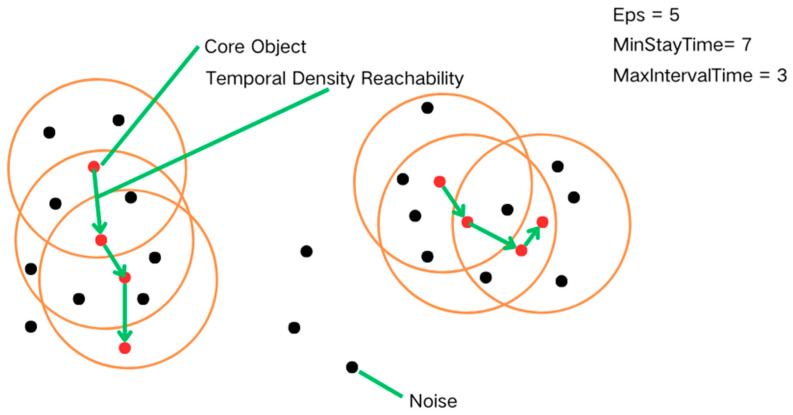
Conceptual explanation diagram of the T-DBSCAN algorithm.

DBSCAN is a spatial clustering algorithm capable of handling noisy data [16]. The principle is to form clusters by dividing regions with sufficient density to recognize clusters of arbitrary shapes in noisy spatial databases. DBSCAN defines a cluster as a maximal set of densely connected points [17]. The T-DBSCAN algorithm is an improved algorithm based on the DBSCAN algorithm, which not only pays attention to spatial density but also introduces “time” as the key clustering algorithm and defines the closeness of clusters by setting the time constraint parameter (Figure 1). The fundamental concepts in T-DBSCAN are as follows:

Suppose the sample set is known, $D=\left\{a_{1},a_{2},\ldots ,a_{m}\right\}$, where each sample,$\;a_{i}\in D,$ represents a point with spatial and temporal properties. Each sample point${\;a}_{i}\;$is of the form$\;a_{i}=\left(x_{i},y_{i},t_{i}\right)$. Included among these,$\;\left(x_{i},y_{i}\right)\;$indicates spatial coordinates and $t_{i}\;$indicates a time stamp. Then, the specific density description of T-DBSCAN is defined as follows:

1. **Spatial Neighborhood (EPS):** Given a spatial radius parameter ϵ for a sample point${\;a}_{i}\in D$, its spatial neighborhood $N_{\epsilon }\left(a_{i}\right)$ is defined as the set of all points that satisfy the following conditions:

(1) $$N_{\epsilon }\left(a_{i}\right)=\{a_{j}\in D|dist\left(a_{i},a_{j}\right)\leq \epsilon \}\;$$

where $dist\left(a_{i},a_{j}\right)$ is the geospatial distance between $a_{i}$ and $a_{j}$. In the algorithm implementation, we use the Haversine formula to calculate it.

2. **Temporal Neighborhood:** A temporal neighborhood $N_{\epsilon }\left(a_{i}\right)$ is a contiguous subset obtained by ordering the spatial neighborhood $N_{\epsilon }\left(a_{i}\right)$ of $a_{i}$ according to the time t. In this subset, the time difference between any neighboring points does not exceed the time threshold parameter MaxIntervalTime. Specifically:

I. First, all points in $N_{\epsilon }\left(a_{i}\right)$ are sorted in ascending order according to time t to obtain the sorted set $N_{\epsilon }^{\prime }\left(a_{i}\right)$.

II. In $N_{\epsilon }^{\prime }\left(a_{i}\right),$ select the largest consecutive subset starting from the first element that satisfies the following condition: For two points, $a_{j}$ and $a_{j+1}$, in the subset, the time difference is $\left|a_{j}-a_{j+1}\right|\leq MaxIntervalTime$.

The subset thus obtained is the temporal neighborhood $N_{t}\left(a_{i}\right)$ of $a_{i}$.

3. **Core Object:** A core object (or core point) is a point for any sample $a_{i}\in D$ if the maximum time difference of the points in its temporal neighborhood $N_{t}\left(a_{i}\right)$ satisfies the minimum residence time threshold MinStayTime, i.e.:

(2) $$\underset{a_{j},a_{k}\in N_{t}\left(a_{i}\right)}{\max}\left|t_{j}-t_{k}\right|\geq \mathrm{minStayTime}$$

then, $a_{i}$ is said to be a core object. This means that the core object must maintain a certain dwell time in the temporal neighborhood to determine temporal continuity and spatial density.

4. **Temporal Density Direct:** $a_{j}$ is said to be temporally density direct from $a_{i}$ if $a_{j}$ lies in the temporal neighborhood $N_{t}\left(a_{i}\right)$ of $a_{i}$ and $a_{i}$ is a core object. Note that the converse does not necessarily hold unless $a_{j}$ is also a core object [18].

5. **Temporal Density Reachability:** for $a_{i}$ and $a_{j}$, $a_{j}$ is said to be reachable by $a_{i}$ temporal density if there exists a sequence of samples${\;q}_{1},q_{2},q_{3},\ldots ,q_{T}$, satisfying $q_{1}=a_{i},q_{T}=a_{j}$, and $q_{t+1}$ is directly reachable by $q_{t}$ temporal density. That is, the temporal density reachable satisfies the transmissibility. At this point, the passing samples $q_{1},q_{2},q_{3},\ldots ,q_{T-1}$ in the sequence are all core objects, because only the core objects can make the other sample’s time density reachable. Note that temporal density reachable also does not satisfy symmetry, and this can be derived from the asymmetry of temporal density directly reachable.

6. **Temporal Density Connectedness:** For $a_{i}$ and $a_{j}$, $a_{i}$ and $a_{j}$ are said to be temporally density connected if there exists a core object sample $a_{k}$ such that both $a_{i}$ and $a_{j}$ are directly connected by temporal density $a_{k}$. Note that the density connectedness relation is symmetry-satisfying. This can be easily derived from the definition of temporal density reachable.

7. **Noise:** Noise points are those sample points that are neither core objects nor part of any cluster. In T-DBSCAN, noise points cannot form clusters with other samples through the relationship of time-density direct or time-density reachable and are, therefore, isolated. In other words, the samples in the temporal neighborhood of a noise point cannot satisfy the requirement of minimum residence time MinStayTime and, therefore, cannot be core objects.

### 2.2. Optimization Strategies for Algorithms

#### 2.2.1. Neighborhood Lookup Optimization Strategy

**Figure 2:**
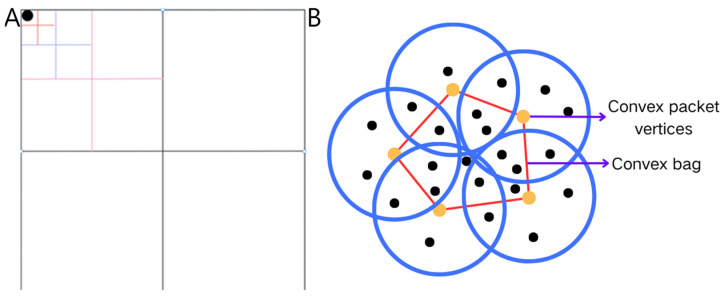
(**A**) Schematic diagram of a quadtree. (**B**) Schematic diagram of bump pack optimization.

The computational efficiency bottleneck of traditional DBSCAN in large-scale avian trajectory data analysis primarily stems from its global traversal-based neighborhood query mechanism, which incurs $O\left(n^{2}\right)$-level time complexity. This study employs a quadtree spatial index mechanism to address this limitation to restructure data organization. As shown in Figure 2A, this method recursively partitions two-dimensional geographical space into sub-regions, establishing a multi-level index structure that captures spatial distribution characteristics [19,20]. This optimization confines neighborhood queries from the global dataset to localized spatial units, leveraging spatial topological relationships to enable rapid unit localization and batch data retrieval, thereby significantly reducing the computational load in massive data environments.

#### 2.2.2. Domain Extension Optimization Strategy

Traditional expansion strategies generate substantial redundant computations in densely populated data regions due to the indiscriminate processing of neighboring points. This study proposes a convex hull boundary-guided expansion strategy to address this issue. As demonstrated in Figure 2B, the Graham scan algorithm [21] is employed to compute the minimum convex hull geometry of target neighborhoods, prioritizing propagation from boundary points located at convex hull vertices. This strategy eliminates redundant computations on interior points while preserving the density-reachable principle, thereby reducing the time complexity of the expansion process. Simultaneously, radial propagation from boundary point clusters ensures the integrity of cluster topology.

The doubly optimized algorithm significantly improves operational efficiency in large-scale datasets while maintaining original clustering accuracy. Subsequent experimental validation will demonstrate the performance of these strategies across diverse datasets to confirm their effectiveness.

### 2.3. Algorithm Logic

**Figure 3:**
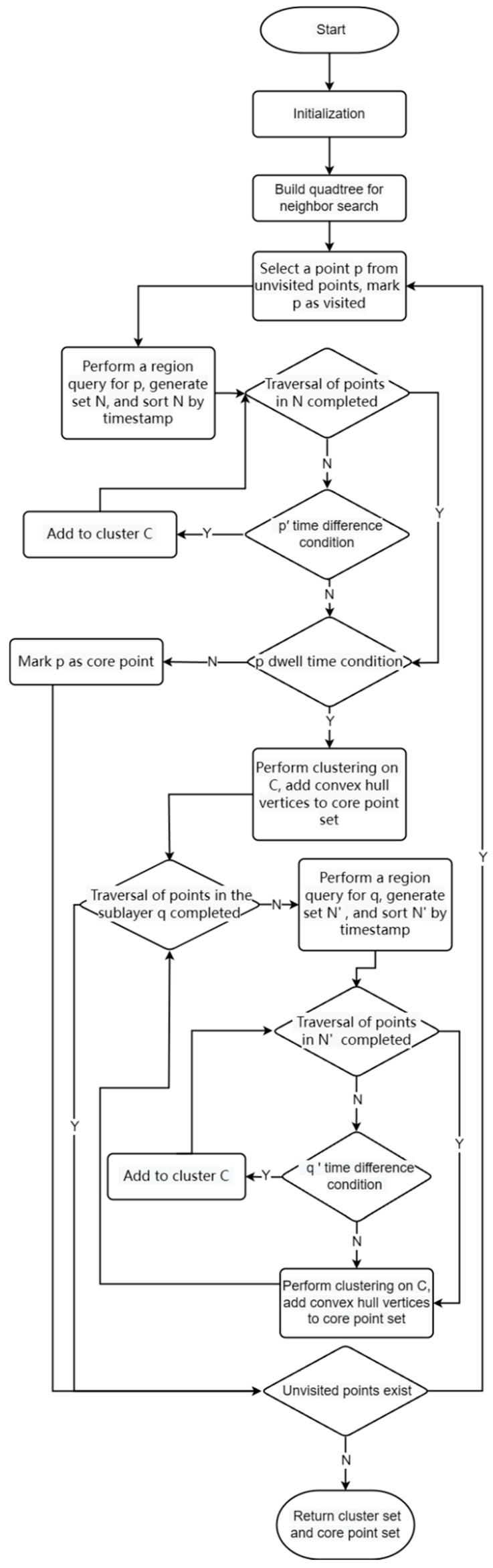
Algorithm flowchart of T-DBSCAN.

Algorithm 1: Time-DBSCAN (T-DBSCAN) is a time and space-based density clustering algorithm. The flowchart of the algorithm is shown in Figure 3.
**Algorithm 1 T-DBSCAN**Input:  Points: an object containing n objects in latitude, longitude, and time.  eps: spatial radius parameter  minStayTime: minimum stay time threshold  maxIntervalTime: maximum time difference parameter  Output: a set of clusters and core points based on temporal and spatial density  Methods: **1.** **Initialization**   1.1 Mark all points as unvisited.    1.2 Set all cluster labels to −1.**2.** **Construct the quadtree**   2.1 Create a quadtree region covering all points.   2.2 Insert all points into the quadtree for efficient neighborhood queries.
**3.** **Clustering process**   3.1 Randomly select an unvisited point p   3.2 Mark p as visited.**4.** **Domain query**   4.1 Query all neighbors of p within eps radius to form set N.
   4.2 Sort N by timestamp.**5.** **Temporal conditional judgment**
   5.1 Mark p as a core point if its maximum dwell time in N is ≥ minStayTime otherwise, mark p as noise.**6.** **Cluster creation and expansion**
   6.1 Create a new cluster *C* and add p to *C*.

   6.2 Sort N by timestamp. 

   6.3 For each point $p^{\prime }$ in N:

   6.4 If $\Delta t\left(p^{\prime },p\right)$ ≤ maxIntervalTime, add $p^{\prime }$ to *C*. 
   6.5 Otherwise, stop expansion.**7.** **Convex hull expansion**
   7.1 Extend the cluster using only convex hull vertices in N.     7.2 Repeat steps 4 and 5 for each convex hull vertex.**8.** **End conditions**
   8.1 If no unvisited points remain, output C.     8.2 If all points are visited, return the set of clusters and core points.

### 2.4. Algorithm Time Complexity Analysis

From the above algorithm flow, the time complexity of the algorithm is analyzed in three main parts: neighborhood query, convex hull computation, and neighborhood expansion. The first part of the neighborhood query uses the spatial data structure of the quadtree. The depth of the quadtree is $O\left(\log n\right)$, where n is the total number of points in the data set. Assuming that there are k points in the neighborhood of the sample point p, the complexity of querying the sample point  p is $O\left(k+\log n\right)$. Since the neighborhood query for each point may return multiple points, the total query complexity for the entire dataset is $O\left(n\log n+kn\right)$, where k is the average number of points in the neighborhood, which is usually much smaller than n. The second part of the convex hull computation, the convex hull algorithm, uses Graham’s scanning method, with a time complexity of $O\left(k\log k\right)$, where k is the total number of points returned in the neighborhood query. The total number of points considered in the dataset, because, in the algorithm, only the points in the neighborhood are used for convex hull calculation, and, in general, k will be much smaller than n, so the time complexity of calculating the convex hull is approximated as $O\left(k\log k\right)$.The third part of the step is the neighborhood expansion. The neighborhood expansion of each core point partially relies on the expansion of the vertices of the convex hull, and the query of the neighboring points still relies on the quadtree for the neighborhood lookup. For each core point, the time complexity of finding the neighborhood points through the quadtree is $O\left(\log n\right)$. These points will then be further processed through convex hull expansion. Thus, the time complexity of expanding the neighborhood is $O\left(k\log k+k\log n\right)$. Here, k is the number of points in the neighborhood. Combining the above three parts, we can approximate the time complexity of the whole algorithm as:

(3) $$O\left(n\log n+k\log k+k\log n\right)$$

where n is the total number of points in the dataset and k is the number of points in the neighborhood, which is usually k ≪n, so the overall complexity of the algorithm will be closer to $O\left(n\log n\right)$, which is much less than the time complexity $O\left(n^{2}\right)$ of DBSCAN [22].

### 2.5. Case Studies

#### 2.5.1. Computing Environment

Conventional DBSCAN algorithm with improved T-DBSCAN algorithm running on an Intel Core i5-1135G7 2.40 GHz, 16 GB RAM laptop.

#### 2.5.2. Bean Goose Satellite Positioning Data

The bean goose (*Anser fabalis*) [23] is a large, migratory waterbird, widely distributed in Eurasia and northern Africa, with two subspecies wintering in China. The lakes and wetlands of the middle and lower reaches of the Yangtze River are the main wintering grounds. It lives in subarctic taiga lakes or subplain forested river valleys during the breeding season. During migration and winter, it lives in open plains grasslands, marshes, reservoirs, rivers, lakes, coastal shores, and adjacent farmlands [24]. This study focused on the bean goose (*Anser fabalis*) as the research subject. A tracking device weighing 50 g, manufactured by Global Messenger Technology Co., Ltd., Changsha, Hunan Province, China, was employed, with its sampling frequency varying depending on voltage, ranging from approximately once per hour to once every four hours. At capture, the individual weighed 3.117 kg, with the tracker accounting for 1.6% of its body weight, adhering to the standard of not exceeding 3% of its body weight [25]. Data collection from December 2022 to December 2024 yielded 16,396 valid entries containing information such as timestamp, longitude, latitude, positional accuracy, activity level, and speed.

### 2.6. Statistical Analysis

#### 2.6.1. Identification Accuracy

The experiments used a multi-layered validation approach to evaluate the recognition accuracy. Specifically, the results of algorithm identification were compared with actual bird habitat data and verified by combining remote sensing data with manual identification of surrounding geographic features. At the same time, domain experts were consulted to assess the reasonableness of the recognition results and to determine whether they conformed to the typical habitat characteristics of the target birds.

#### 2.6.2. Algorithm Running Rate

To evaluate the running efficiency of the algorithm, the same multiple datasets are used to record the running time of the algorithm and compare the running time of the algorithm under different-sized datasets.

#### 2.6.3. Calinski-Harabasz (CH) Metrics

To assess the cluster dispersion of clustering algorithms, the metrics for evaluating the clustering effectiveness of density clustering algorithms are used. The CH index [26,27] evaluates the inter- and intra-cluster dispersion, which is defined as:

(4) $$\mathrm{CH}=\frac{\sum_{j=1}^{k}n_{j}\cdot |\bar{x_{j}}-\bar{x}{|}^{2}/\left(k-1\right)}{\sum_{j=1}^{k}\sum_{x\in C_{j}}|x-\bar{x_{j}}{|}^{2}/\left(n-k\right)}$$

where n denotes the total number of samples, k is the number of clusters, $n_{j}$ denotes the number of samples in cluster $c_{j}$, $\bar{x_{j}}$ is the center of mass of cluster $c_{j}$, $\bar{x}$ is the center of mass of the entire dataset, $|\bar{x_{j}}-\bar{x}{|}^{2}$ measures the sum of the squares of the distances between the center of mass of cluster $c_{j}$ and the center of mass of the whole (inter-cluster scattering), and $|x-\bar{x_{j}}{|}^{2}$ measures the squares of the distances between each sample point p and the center of mass of the clusters in cluster $c_{j}\;$(intra-cluster scattering). Higher values of CH indicate better clustering, i.e., the data are more tightly packed in clusters, and inter-cluster data are more dispersed.

## 3. Results

### 3.1. Characteristics of Satellite Tracking Data for Bean Goose

**Figure 4:**
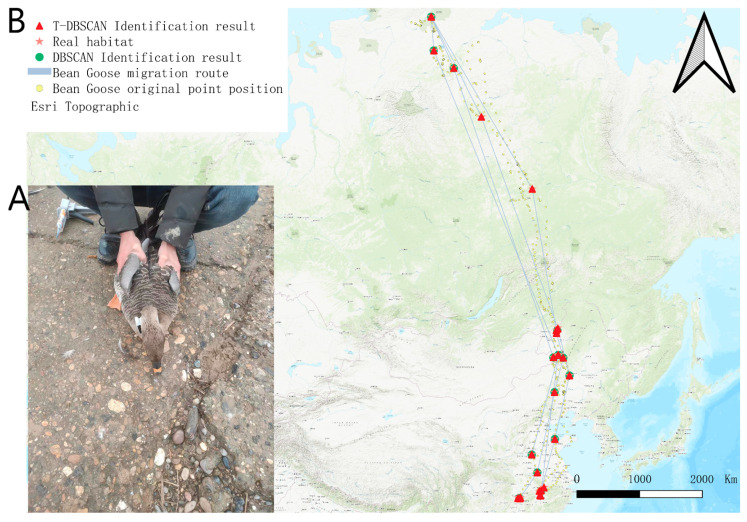
Bean goose satellite positioning tracking. (**A**) Satellite tracker installation. (**B**) Satellite tracking location display map.

The wild goose was captured and installed with satellite tracking on 27 December 2022 in the Dongting Lake area (Figure 4A). A total of 16,395 valid tracking points were obtained from the satellite tracking data of the target wild goose. The tracking time was from 27 December 2022 to 11 December 2024, which covered the migratory and stopover information of the target wild goose in many geographic areas. The dataset records two complete migration cycles of the bean goose, fully demonstrating its wide migratory range and its choice of key stopover sites during migration. In terms of habitat distribution, the breeding site of the target bean goose was located in the Taimel Nature Reserve (73°47′00.2″ N, 97°47′11.8″ E), a water-rich and ecologically suitable high-latitude area, which provides ideal conditions for bean geese to breed. In terms of wintering grounds, the target bean goose is mainly found in the Dongting Lake basin (28°45′10.8″ N, 112°47′45.6″ E) and the farmland area near the Yellow River (35°03′51.8″ N, 114°50′09.6″ E). These areas are characterized by mild climate and abundant food resources, which provide superior survival conditions for bean geese to overwinter.

Figure 4B shows the migration trajectory, original tracking points, and T-DBSCAN algorithm results of the target goose. During the first northward migration (4 March 2023 to 31 May 2023), the target bean goose traveled through six stopover sites with stopover times of 14, 18, 7, 9, 16, and 7 days, demonstrating significant stopover diversity during migration. The first southward migration (15 September 2023–8 November 2023) passed through three stopover sites with stopover times of 12, 9, and 24 days, reflecting more concentrated habitat use during fall migration. During the second northward return (11 March 2024–2 June 2024), the target bean goose stopped at four sites with stopover times of 8, 11, 24, and 7 days, respectively, and migratory behaviors tended to become more regular. The second southward migration (5 September 2024 to 4 November 2024) passed through two stopover sites with stopover times of 14 and 24 days, showing a more significant concentration of stopovers in the later stages of the migration.

### 3.2. Impact of Optimization Strategies

#### 3.2.1. Impact of Neighborhood Search

The experimental results run from Table 1 show that the quadtree optimization significantly improves the running efficiency of the T-DBSCAN algorithm. The algorithm’s runtime is reduced by more than 50% at the size of the location dataset of more than 10,000 loci. In addition, the number of clusters remains consistent, and the recognition difference rate is around 0.1%, indicating that the quadtree optimization improves the efficiency without affecting the algorithm’s accuracy.

#### 3.2.2. Impact of Neighborhood Extension

The convex hull optimization strategy effectively reduces computational complexity in neighborhood expansion through geometric feature screening. The algorithm sorts neighborhood point sets via polar angle ordering criteria, constructs minimal convex hull structures using Graham’s scanning algorithm, systematically eliminates non-critical interior points, and retains boundary vertices as candidate point sets for expansion operations. This mechanism optimizes traditional full-range expansion into targeted key-point expansion through geometric boundary feature recognition, significantly mitigating redundant computations in high-density regions. As evidenced in Table 2, the algorithm’s operational efficiency demonstrates substantial improvement after implementing convex hull optimization, validating the strategy’s effectiveness in preserving clustering structural integrity while optimizing computational resources.

### 3.3. The Comparison of Stopover Site Identification

**Figure 5:**
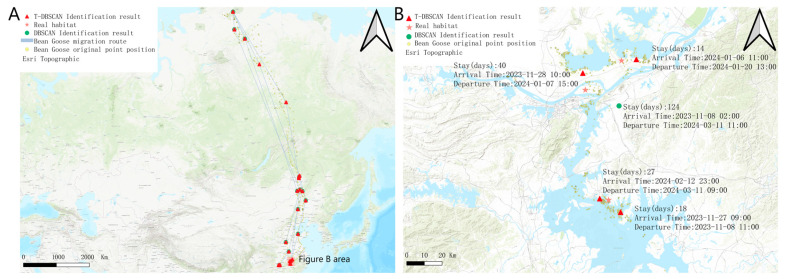
Demonstration of habitat distribution. (**A**) Results of DBSCAN and T-DBSCAN on the map under bean goose data. (**B**) Results of the two algorithms for the same area (longitude 115.84° to 116.66°, latitude 29.01° to 30.05°).

Figure 5 shows the results of the two algorithms under the bean goose dataset, and the performance differences between the two algorithms under the bean goose dataset can be observed. The stopover site distribution recognized by the T-DBSCAN algorithm is closer to the real ecological distribution of the habitat. It can restore the migration path of the target bean goose as well as the location of the stopover sites. In contrast, although DBSCAN can also provide a more accurate spatial distribution, it fails to consider the temporal factor, and its results tend to aggregate data from multiple periods into a single stopover site, making it difficult to accurately reveal spatial and temporal dynamics over many years (Figure 4A).

The difference in performance between the two algorithms when dealing with multi-year data is further highlighted in Figure 5B. T-DBSCAN, in conjunction with temporal parameters, can recognize patterns of stays at the same geographic location across years and accurately calculate the duration of each stay. This optimization enables T-DBSCAN to better reflect the spatiotemporal behavioral characteristics of migratory species, providing a scientific basis for studying multi-year ecological dynamics.

### 3.4. Effectiveness Evaluation of T-DBSCAN

#### 3.4.1. Stopover Site Identification Accuracy

**Figure 6:**
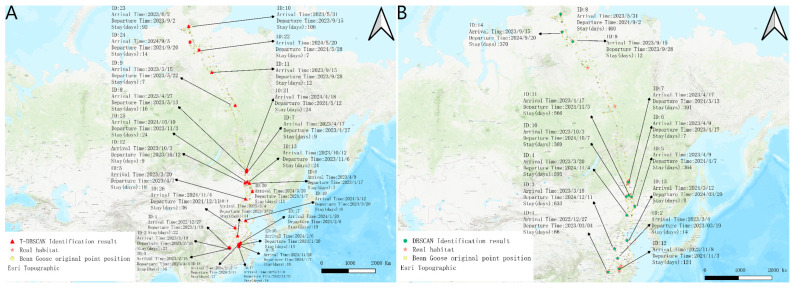
The results of the two algorithms in the bean goose data set. (**A**) Results of the T-DBSCAN. (**B**) Results of the DBSCAN.

Figure 6 shows the habitat identification results of DBSCAN and T-DBSCAN algorithms in the bean goose dataset. T-DBSCAN almost doubles the number of habitats identified compared to the DBSCAN algorithm, with 14 stopover sites overlapping in the results of both algorithms. In addition, T-DBSCAN identified an additional 9 stopover sites within the geographic areas identified by DBSCAN and added 3 new geographic areas not identified by DBSCAN. This shows that T-DBSCAN’s performance in stopover site identification is more comprehensive and accurate.

Further, combined with the ecological context analysis, it can be found that the bean goose mainly inhabited areas such as rivers, lakes, and their surrounding wetlands and often foraged in farmland and plains during migration (Tables S1 and S2). Combined with Google maps and ecological data analysis, the latitude and longitude of numbers 1, 2, and 3 in Figure 6A (between 112.484° and 112.92° longitude and 28.753° and 28.9° latitude) are located at the junction of the plains’ farmland and lakes, which reflects that the bean goose frequently roosted and stayed in this area from winter to early spring, and this area has also become the most important area for the bean goose to forage in the area from 27 December 2022 to 4 March 2023 before the bean geese returned north. In contrast, although the DBSCAN algorithm could identify roosting sites more accurately, it failed to take into account the time dimension and, therefore, could not accurately distinguish the distribution characteristics of geographically similar stopover sites at different times. For example, numbers 1, 2, and 3 in Figure 6A were identified as a single habitat (number 1 in Figure 6B), and the algorithm failed to calculate stopover onset time effectively. In contrast, T-DBSCAN could more accurately identify the ecological characteristics of these stopover sites by introducing a temporal parameter, thus revealing their spatiotemporal dynamics and providing higher ecological accuracy.

In addition, the T-DBSCAN algorithm performs better in multi-year data processing. For example, number 14, identified by DBSCAN in Figure 6B, and number 24, identified by T-DBSCAN in Figure 6A, are located in similar geographic locations, and both are important stopover sites for bean geese on their southward migration. Despite the similarity of their spatial locations, T-DBSCAN was able to reveal the actual stopping pattern of the bean goose more accurately than DBSCAN by taking into account the stopping start time and stopping duration. This indicates that T-DBSCAN can effectively capture short-term stopover and long-term roosting areas in migration paths, improving stopover site identification accuracy and providing a more reliable reference for ecological stopover site conservation.

#### 3.4.2. Algorithmic Run Rate

**Figure 7:**
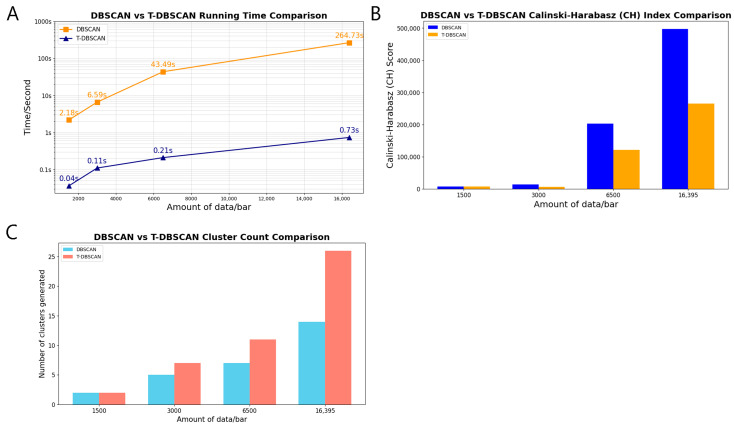
Compared with the DBSCAN algorithm, the T-DBSCAN algorithm has greater efficiency and accuracy. (**A**) Comparison of the time consumption of the two algorithms. (**B**) CH metrics for clustering effects. (**C**) Number of clusters generated by clustering.

Figure 7A shows the comparison of the running rates of the DBSCAN algorithm and the T-DBSACN algorithm with different sizes of bit-point datasets, and the *y*-axis is on a logarithmic scale due to the large difference in running time. With the increase in dataset size, the elapsed time of DBSCAN grows exponentially. At the same time, the T-DBSCAN maintains a smoother rise, showing its significant advantages in neighbor node querying and neighborhood expansion, which equips it to run on large datasets with higher processing efficiency.

#### 3.4.3. Calinski–Harabasz (CH) Indicator Analysis

As seen in Figure 7B,C, under a small dataset, the number of clusters generated by the DBSCAN and T-DBSCAN algorithms is the same, and the CH metrics of T-DBSCAN are higher than those of DBSCAN. As the size of the dataset increases, the number of clusters generated by T-DBSCAN is significantly more than that of DBSCAN, and, according to the Calinski–Harabasz formula [28], shows that when the number of clusters is small, the inter-cluster distance is more likely to remain large, resulting in a relatively higher CH metric score for DBSCAN compared to T-DBSCAN. The significantly larger number of clusters reflects that the algorithm is more detailed in identifying regions with large density variations, which indicates that T-DBSCAN is better than DBSCAN in terms of spatial clustering accuracy. At the same time, although the CH metrics decrease slightly due to the increase in the number of clusters compared to DBSCAN, T-DBSCAN still maintains a high level, which suggests that the algorithm can effectively maintain good clustering performance when dealing with large-scale datasets. Effectively maintains a good clustering structure.

In conclusion, the improved T-DBSCAN algorithm based on DBSCAN can significantly improve habitat identification accuracy, operation efficiency, and clustering structure, especially for large-scale datasets with significant spatial and temporal characteristics.

## 4. Discussion

### 4.1. Algorithmic Implications of Optimization Strategies

Based on the results in Table 1 and Table 2, the algorithm achieved significant efficiency improvements through dual-strategy optimization. Specifically, combining two optimization strategies enabled the T-DBSCAN algorithm to attain an approximately 20-fold improvement in operational efficiency on small-scale datasets, with anticipated even more substantial optimization effects on larger-scale datasets.

**Figure 8:**
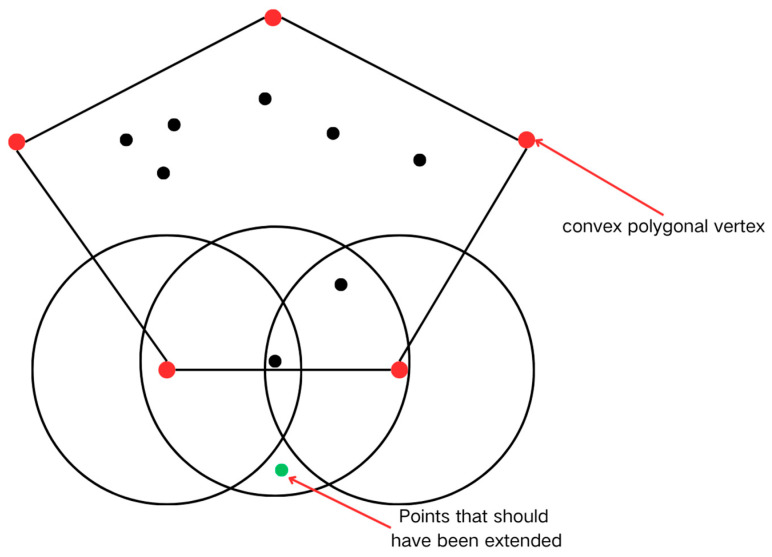
Defects in the bumper.

However, the geometric constraint mechanisms in the convex hull optimization strategy may introduce localized identification biases. As illustrated in Figure 8, the systematic exclusion of non-convex hull points during vertex screening might lead to the omission of specific neighborhood points in complex boundary regions, resulting in minor deviations in some clustering outcomes. Table 2 demonstrates an identification discrepancy rate of approximately 1%, indicating a limited impact on overall clustering accuracy while preserving the integrity of core habitat area determinations.

Future research should enhance the synergy between geometric screening and expansion mechanisms. Potential refinements include modifying vertex selection criteria through adaptive convex hull construction strategies informed by local density distributions and incorporating machine learning algorithms to dynamically tune the vertex selection process, improving adaptability and performance.

### 4.2. The Significance of Habitat Management for Migratory Bird Conservation

Habitat management is a key component of migratory bird conservation. Different management strategies have played a significant role in improving the living environment of migratory birds. For example, Zuo, A. and other researchers [29] implemented three management tools, namely, fire, mowing, and plowing, and conducted on-site monitoring of the overwintering geese habitat in Dongting Lake. These measures effectively promoted the growth of short-tipped sedge, provided stable and high-quality food resources for overwintering geese, and thus improved the quality of the habitat and the reproductive success and survival rate of overwintering geese. Similarly, since 2013, the Ecological Project of Chongming [30] for bird habitat optimization successfully curbed the spread of the exotic species *Spartina alterniflora* Loisel, bringing the number of wintering waterbirds to more than 24,000 in 2016, further highlighting the importance of protecting the ecology of native wetlands and preventing the invasion of exotic plants for the conservation of migratory birds. Dong Li et al. [31] used satellite tracking and remote sensing technology to identify the habitat environment, clarified the migratory paths and stopover sites of migratory birds, provided valuable first-hand information for migratory bird conservation, solved many habitat research and monitoring problems that were difficult to conduct on-site surveys, and enhanced the real-time nature of habitat research.

Looking ahead, the integrated framework combining the T-DBSCAN spatiotemporal clustering algorithm with satellite tracking technology proposed in this study can provide dynamic and refined decision-making support for habitat management by analyzing the spatiotemporal heterogeneity of bird migration. Specifically, the T-DBSCAN algorithm can precisely identify the critical time windows during which migratory birds arrive at specific geographic regions. This information enables protected areas to implement targeted conservation measures during these key periods. For instance, regional logging bans or agricultural restrictions can be enforced within the identified habitation time windows to minimize human disturbance, avoid disrupting the habitats of migratory birds, and ensure their regular migration.

Furthermore, the algorithm can detect newly emerging potential habitats (e.g., wetland patches formed due to climate warming). For such areas, ecological restoration projects such as reforestation and wetland substrate restoration can gradually be prioritized to transform them into stable resource supply zones. This approach strengthens the protection of critical habitats and establishes a foundation for the sustainable management of long-term habitats. Protected areas can adjust conservation strategies based on changes in bird residence times to ensure habitat effectiveness and stability.

On the other hand, if the algorithm reveals significant reductions in residence duration (e.g., from an annual average of 20 days to 5 days) or declines in flocking intensity at historical habitats, it may indicate ongoing vegetation degradation, resource depletion, or anthropogenic damage in these regions. Protected areas can then prioritize monitoring and intervention to mitigate further environmental deterioration. Thus, the application of the T-DBSCAN algorithm not only uncovers changes in migratory bird habitats but serves as an early warning system for ecological shifts.

From a broader perspective, this technology enhances the spatiotemporal precision of conservation actions while advancing management paradigms from experience-driven approaches to model-assisted decision-making. Uncovering hidden correlations between avian behavior and microenvironmental factors drives habitat protection toward more efficient and intelligent practices.

### 4.3. Limitations and Future Directions

Although this study offers a novel methodological perspective for avian migration ecology through individual-scale tracking analysis, it should be noted that the algorithmic framework still shares the common parameter sensitivity issue inherent to the traditional DBSCAN algorithm. Crucially, the ecological rationality of parameter settings directly influences the validity of habitat identification outcomes—researchers must perform biological validation and calibration of algorithmic parameters based on the migratory ecological traits of target species (e.g., stopover site residency duration, daily movement ranges). Prior ecological knowledge can also constrain parameter ranges; for instance, the 25 km spatial threshold and 3-day temporal window proposed in this study may serve as reference criteria for parameter selection in species trajectory analyses.

Existing studies [32,33,34] reveal significant migratory route variations among conspecific individuals, highlighting the need for cautious interpretation when applying this method to non-colonial species. Single-individual tracking data may reflect idiosyncratic behavioral preferences rather than species-level habitat utilization patterns. This study focuses on the bean goose (*Anser fabalis*), a colonial migratory waterfowl whose individual movement trajectories exhibit high population-level behavioral consistency. A multi-scale validation framework was implemented to mitigate identification biases arising from individual behavioral variability. Results from Supplementary Table S2 demonstrate a 100% accuracy rate in identifying actual habitats for studied individuals, confirming the ecological validity of this method for colonial species habitat recognition.

Future research should enhance the findings’ generalizability and ecological robustness by expanding sample sizes to include individuals of diverse sexes, ages, and migratory histories while integrating external environmental factors. Additionally, developing an ecologist-oriented parameter optimization framework—via automated calibration modules—could reduce reliance on prior knowledge and improve methodological transferability in cross-species studies.

## 5. Conclusions

In this paper, the limitations of the traditional DBSCAN algorithm in bird migration trajectory clustering are improved. Since the DBSCAN algorithm only relies on spatial neighborhood and minimum neighborhood points and ignores the time dimension, it is difficult to identify similar stopover sites in different periods accurately, and, at the same time, it is less efficient to run under large-scale datasets. Therefore, this paper proposes the Time-DBSCAN (T-DBSCAN) algorithm, which replaces the minimum number of neighborhood points with the minimum stopover duration. The algorithm combines spatial distance and temporal distance to optimize the neighborhood query using a quadtree spatial index. It introduces a convex hull optimization strategy to reduce the number of expansion nodes for the redundant computation problem in neighborhood expansion, which makes the expansion process more oriented and critical. Although convex hull optimization significantly improves the processing efficiency of large-scale data, it still brings some accuracy loss. Future research will focus on improving the convex hull vertex selection strategy and developing new algorithms to capture better ecological information related to habitat changes to further enhance the accuracy and application of bird habitat identification.

## Figures and Tables

**Table 1:** Test results with and without quadtree optimization under different datasets.

Arithmetic	Norm	1500 Points	3000 Points	6500 Points	16,495 Points
T-DBSCAN (without quadtree optimization)	Time (ms)	639.975	1983.07	9380.1	52,829.7
	Number of clusters	2	7	11	26
T-DBSCAN (with quadtree optimization)	Time (ms)	658.41	1129.59	4096.93	13,355.5
	Number of clusters	2	7	11	26
	Recognition difference rate	0	0	0	0

**Table 2:** Test results with and without convex hull optimization under different datasets.

Arithmetic	Norm	1500 Points	3000 Points	6500 Points	16,495 Points
T-DBSCAN (convex hull-free optimization)	Time (ms)	658.096	1145.87	4083.28	13,431.6
	Number of clusters	2	7	11	26
T-DBSCAN (with convex hull optimization)	Time (ms)	36.36	109.81	209.796	732.341
	Number of clusters	2	7	11	26
	Recognition difference rate	0	0.0017	0.0023	0.0137

## Data Availability

Data are contained within the article and Supplementary Materials.

## Supplementary Materials

The following supporting information can be downloaded at: https://www.mdpi.com/article/10.3390/biology14030277/s1, Table S1. Habitat identification table for DBSCAN in the bean goose dataset; Table S2. Habitat identification table for T-DBSCAN in the bean goose dataset.
