# Supplementary material for: The T-DBSCAN Algorithm for Stopover Site Identification of Migration Birds Based on Satellite Positioning Data
Source: Biology (Basel). 2025 Mar 7;14(3):277. doi: 10.3390/biology14030277 (PMC11940076; doi:10.3390/biology14030277)
Supplement: Supplementary file 1 [file biology-14-00277-s001.zip › biology-3488201-supplementary.pdf]

Table S1. Habitat identification table for DBSCAN in the Bean Goose dataset.

No.	Arrival Time	Departure Time	Stay (days)	Longitude	Latitude	Description	Habitat or not
1	2022/12/27 15:00	2023/3/4 2:00	66	112.72	28.80	Rivers Minor State	Yes
2	2023/3/4 9:00	2023/3/19 9:00	14	115.78	32.54	Plains Farmland Rivers	Yes
3	2023/3/19 12:00	2024/12/11 14:00	633	114.83	35.06	Plains Farmland Rivers	Yes
4	2023/3/20 1:00	2024/11/4 6:00	595	118.69	43.24	river valley	Yes
5	2023/4/9 2:00	2024/4/7 8:00	364	121.23	45.26	Plains Farmland Rivers	Yes
6	2023/4/9 7:00	2023/4/17 7:00	7	120.17	47.33	Haraha River	Yes
7	2023/4/17 14:00	2024/5/13 3:00	391	119.15	50.40	Argun River	Yes
8	2023/5/31 15:00	2024/9/2 20:00	460	97.79	73.77	Plains, riverine areas	Yes
9	2023/9/15 19:00	2023/9/28 1:00	12	101.61	71.16	Plains, lake areas	Yes
10	2023/10/3 9:00	2024/10/7 6:00	369	118.49	47.37	lochs	Yes
11	2023/4/17 8:00	2024/11/3 17:00	566	119.33	47.66	lochs	Yes
12	2023/11/8 2:00	2024/3/11 11:00	124	116.32	29.70	plains farmland	Yes
13	2024/3/12 4:00	2024/3/20 18:00	8	118.74	37.19	plains farmland	Yes
14	2023/9/15 15:00	2024/9/20 2:39	370	98.21	72.10	plains and lakes	Yes

Table S2. Habitat identification table for T-DBSCAN in the bean goose dataset.

No.	Arrival Time	Departure Time	Stay (days)	Longitude	Latitude	Description	Habitat or not
1	2022/12/27 15:00	2023/01/19 10:00	22	112.484	28.8126	Hilly region, near small rivers	Yes
2	2023/01/19 11:00	2023/02/16 07:00	27	112.796	28.753	Hilly terrain, close to forest edges	Yes
3	2023/02/15 10:00	2023/03/04 02:00	16	112.911	28.8986	Small valley area with mixed vegetation	Yes
4	2023/03/04 09:00	2023/03/19 09:00	14	115.782	32.5464	Jianghuai Plains, fertile agricultural land	Yes
5	2023/03/20 01:00	2023/04/07 06:00	18	118.690	43.2466	Low hills near river valleys	Yes
6	2023/04/09 07:00	2023/04/17 07:00	7	120.178	47.3347	Low mountain region with scattered rivers	Yes
7	2023/04/17 16:00	2023/04/27 05:00	9	119.040	50.0905	Plateau and mountain region, spring habitat	Yes
8	2023/04/27 07:00	2023/05/13 07:00	16	118.992	50.4077	Hilly terrain, near seasonal water bodies	Yes
9	2023/05/15 05:00	2023/05/22 07:00	7	114.908	63.3306	Russian tundra, early summer habitat	Yes
10	2023/05/31 17:00	2023/09/15 05:00	106	97.8059	73.7739	Polar tundra, consistent summer foraging area	Yes
11	2023/09/15 20:00	2023/09/28 01:00	12	101.617	71.1634	Siberian Highlands, transitional migration	Yes
12	2023/10/03 09:00	2023/10/12 17:00	9	118.489	47.3989	High/altitude areas with dense vegetation	Yes

13	2023/10/12 18:00	2023/11/06 13:00	24	119.319	47.6794	Mountain region near freshwater sources	Yes
14	2023/11/08 11:00	2023/11/27 09:00	18	116.338	29.1745	Yangtze River mid/lower reaches, wintering region	Yes
15	2023/11/28 10:00	2024/01/07 15:00	40	116.119	29.8746	Yangtze River plains, adjacent to wetlands	Yes
16	2024/01/06 11:00	2024/01/20 13:00	14	116.429	29.943	Wetlands along Yangtze River tributaries	Yes
17	2024/01/20 14:00	2024/02/08 15:00	19	116.907	30.3616	Lake area in the lower Yangtze River	Yes
18	2024/02/12 23:00	2024/03/11 09:00	27	116.217	29.2429	Yangtze floodplain, stable stopover site	Yes
19	2024/03/12 04:00	2024/03/20 18:00	8	118.749	37.190	Farmland and small river confluences	Yes
20	2024/03/26 09:00	2024/04/07 08:00	11	121.233	45.2601	Northeastern Plains, close to marshlands	Yes
21	2024/04/18 17:00	2024/05/12 20:00	24	119.258	50.5854	Plateau region with scattered river systems	Yes
22	2024/05/20 19:00	2024/05/28 05:00	7	106.290	68.3101	Siberian tundra, short/term summer habitat	Yes
23	2024/06/02 19:00	2024/09/02 20:00	92	97.7866	73.7834	Polar tundra, consistent annual summer site	Yes
24	2024/09/05 06:00	2024/09/20 02:39	14	98.2554	72.0965	Polar ice field, short transitional stay	Yes
25	2024/10/10 09:00	2024/11/03 17:00	24	119.341	47.649	Hilly terrain, abundant autumn resources	Yes
26	2024/11/04 20:00	2024/12/11 14:00	36	114.836	35.0644	Yellow-Huai River plains, wintering habitat	Yes
